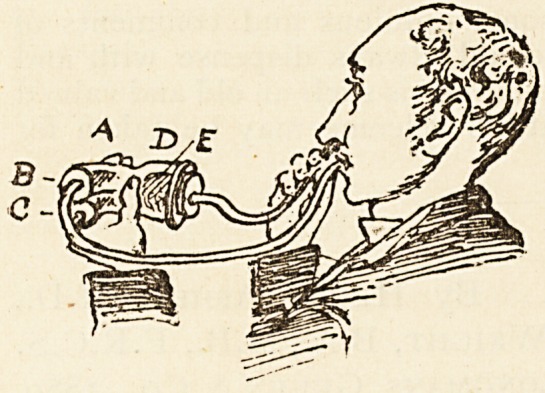# Notes on Preparations for the Sick

**Published:** 1890-03

**Authors:** 


### Notes on Preparations for the Sick.

### A New Instrument for Ammonium Chloride and other Vapours.

Dr. Fred. A. A. Smith, Hon. Surgeon Cheltenham Eye, Ear, and Throat Infirmary, sends us the following note: "In all the inhalers with which I am acquainted, the fumes are generated by drawing them through the mouth and exhaling them through the nose. For the fumes to reach the middle ear, the easiest method in most cases is by Valsalva's plan. The above instrument acts perfectly; all that is necessary being to insert the nasal rubber end into one nostril, and gently blow through the mouthpiece. The fumes will then be generated, and enter the one nostril and return by the other. For the fumes to enter perfectly into the Eustachian tubes and middle ear, the patient must be told to intermittently close the open nostril, when the fumes will be felt in the middle ear. By taking away the ammonium chloride sponges, any other vapours may equally well be used by dropping the medicines on the sponge in enlarged part D. The air inhaled, coming from the warm lung, is far preferable to the cold external atmosphere. If instead of the rubber nose end, one be made to fit the Eustachian catheter, the fumes can easily be made to enter the Eustachian tubes where catheterism is deemed necessary. Messrs. Mayer and Meltzer, 71 Great Portland Street, London, W., have made the above entirely to my satisfaction, and can supply the same."

**Morrhuol Chapoteaut. Vin de Chapoteaut. Dialysed Pepsine, in pearls. Santal-midy, in capsules. Valerianic Ether, in capsules. Joy's Asthma Cigarettes. Dessicated Malt Extract.**—Wilcox & Co., London.

These have been forwarded to us by Messrs. Wilcox and Co , as samples of some of the new remedies prepared by Messrs. Rigaud and Chapoteaut, of Paris. They appear to be elegant and useful preparations, carefully prepared, and reliable.

We have found that patients who cannot take or digest cod liver oil in other forms, can swallow the **Morrhuol Pills**, and gain weight in consequence.

The **Wine** is a liquid peptone, containing the pure peptone of ten grammes of beef in each wineglassful.

The **Asthma Cigarettes** are too well known to need any description or commendation.

The advantages of the **Desiccated Malt Extract** are: first, its richness in diastase, through being prepared *in vacuo* at a temperature of 100° Fahrenheit; and, secondly, the impossibility of its fermenting, owing to being in a powder; whereas with the semi-fluid extracts the merest trace of fermentation (and they are seldom free from such) will not only destroy the diastasic properties, but frequently cause diarrhœa, &c. This powder is also far more convenient to take, as it may be either eaten between bread and butter, or dissolved in a liquid.

---

**Peptone of Meat. Peptonate of Iron.** Denaeyer's Peptones Company, London.

This **Peptone of Meat** is a fluid, slightly gelatinous at low temperatures, clear, pale yellow, with an agreeable aroma and a pleasant flavour similar to that of strong beef-tea. It has a specific gravity of 1070 at 15° Cent. The total weight of ash is 3.460 per cent.; that of the solids when dried is 16.96 per cent. It contains no albumen; but the nitrogenous substances, albuminose, peptone, and other extractives amount to 71 per cent. of the dried residue. It is completely sterilised, and keeps fairly well even after the cork has been removed.

Its appearance, flavour, and permanence are superior to any other of the numerous peptones we have examined; patients take it without protest, and ask for more.

We have found that it dialyses readily, and does not contain any deleterious preservative agent; it should therefore be of great utility in all disorders where the gastric and pancreatic digestive powers are deficient.

The **Peptonate of Iron** has a density of 1040 at 15° C., and contains in 100 parts—

| | |
|---|---|
| Pure peptone ... ... ... ... | 6.80 |
| Hydrated oxide of iron ... ... ... | 3.00 |

It is believed to be a definite chemical combination in which the iron is contained in a form eminently suited to the irritable condition of the gastro-intestinal tract in many anæmic dyspeptics. It may also be used for hypodermic injection, when this method is requisite. Theoretically, it should be easy to produce hæmoglobin from such nutriment as this; and probably a preparation of this kind is fully equal to hæmoglobin itself given as medicine.

We have always found it difficult to induce patients to swallow the various preparations of which hæmoglobin is the principal constituent; but this fluid is quite pleasant in appearance and flavour, and patients take it without difficulty. Its hæmatinic value must be comparatively feeble, as is that of hæmoglobin and of dialysed iron: many other preparations of iron cure anæmia much more quickly; but in chronic cases where the digestive power is weak, and where other preparations of iron do not agree, such a compound as this should be of great clinical value.

---

**Liq. Podophyllin et Belladonna c̄ Strychnia. Liq. Podophyllin et Pepsin.**—Hockin, Wilson & Co., London.

These preparations are additions to the Liq. Podophyllin (Hockin) previously mentioned by us in Vol. VI. We there directed attention to the fact that this solution of podophyllin has little or no aperient action, and therefore is likely to be useful when the cholagogue action of the drug is needed and an aperient is not desirable. The addition of belladonna and strychnia in the one fluid must materially increase its activity, and the combination with pepsine ought to be of great value to the bilious dyspeptic. Both fluids should be of considerable utility in many cases of atonic dyspepsia: they are not disagreeable, and are freely miscible with water. We consider them to be a useful addition to the numerous agencies in common use for the treatment of a class of conditions needing a great variety of drugs and varied preparations of the same.

---

**Gelatine-Coated Oval Pills**.—Wyleys & Co., Coventry.

These pills are a great improvement on the older methods of pill-making. They are equal in appearance to any we have seen, and the oval shape is similar to that we have been familiar with as the "McK. and R. Pills."

It would be a great advantage to pill-takers if such pills as these could always be obtained without delay: no one will endure the soft powdered pills as they are still commonly dispensed after having once taken a modern gelatine-coated pill either round or oval.

---

## Figures and Tables

**Figure f1:**